# Mechanosensing at the endoplasmic reticulum by IRE1

**DOI:** 10.21203/rs.3.rs-10144286/v1

**Published:** 2026-07-01

**Authors:** Michaela M. Mayr, Luiz F. Garcia-Souza, Murphy McDermott, Utku Horzum, Yannick Frey, Margot Haun, Stephan Geley, Chao Jiang, Mattias Luber, Anush Bakunts, Camilla Pirolo, Alesia Yakimchyk, Klaus R. Liedl, Wylie Stroberg, Timo Betz, Liam J. Holt, Abdou Rachid Thiam, Hesso Farhan

**Affiliations:** 1 Institute of Pathophysiology, Medical University of Innsbruck, Innsbruck, Austria.; 2 Laboratoire de Physique de l'École Normale Supérieure, Université PSL, CNRS, Sorbonne Université, Université de Paris, Paris, France.; 3 Institute for Systems Genetics, New York University Langone Medical Center, New York, New York 10016, USA.; 4 Third Institute of Physics - Biophysics, Georg-August-Universität Göttingen, Friedrich-Hund-Platz 1, 37077 Göttingen, Germany.; 5 Division of Genetics and Cell Biology. Università Vita-Salute San Raffaele and IRCCS Ospedale San Raffaele, Via Olgettina 58, Milan, Italy.; 6 Department of General, Inorganic and Theoretical Chemistry, University of Innsbruck, Innsbruck, Austria.; 7 Department of Mechanical Engineering, University of Alberta, Edmonton, Alberta, Canada.

## Abstract

Sensing and integration of mechanical forces in eukaryotic cells have largely been attributed to the plasma membrane and the nucleus. Here, we identify the endoplasmic reticulum (ER) as an autonomous mechanosensitive organelle and uncover IRE1 as an ER-resident mechanosensor. We show that applying mechanical forces to ER membranes increases lateral tension, which is sensed by the transmembrane domain of IRE1. Mechano-activation of IRE1 was unrelated to its canonical role in the unfolded protein response and occurred independently of nuclear mechanosensing. Instead, mechanically activated IRE1 triggered JNK signaling and increased global protein synthesis independently of XBP1 splicing. In engineered skeletal muscle tissue, both electrical stimulation and passive stretch similarly activated IRE1, increased translation, and contributed to training-induced increases in contractile force. Collectively, our results uncover a non-canonical role for IRE1 as an ER-based mechanosensor that couples mechanical forces to the regulation of protein translation.

In a dynamic environment, cells must sense and respond to mechanical stimuli to maintain homeostasis and carry out their functions. To interpret these cues, cells employ mechanosensors, proteins that translate mechanical stimuli into biochemical signals, to trigger adaptation. Effective sensing of mechanical signals requires mechanosensors to reside in cellular structures that are affected in morphology and function by mechanical stress.

While it is well established that the plasma membrane and nucleus are primary sites for mechanotransduction^1–3^, only recent work has revealed that other intracellular membranous organelles play relevant parts in mechanosensing and mechanotransduction^4^.

Emerging evidence suggests an important role of the endoplasmic reticulum (ER) in mechanobiology: The ER is the largest cellular organelle, spans the entire cytoplasmic area and forms contacts with many other organelles, the plasma membrane, and the cytoskeleton. We recently showed that tensile stress increases the rate of transport from the ER to the Golgi^5^. Furthermore, changes in cellular contractility, matrix stiffness, or membrane curvature have been shown to alter ER function^6–9^. Crosstalk between the integrity of the ER and nuclear mechanosensing was also recently demonstrated^10^. Finally, the force-bearing protein NOMO1 was recently shown to regulate ER morphology^11^. Thus, the ER has the potential to act as a mechanosensitive platform, but the molecular mechanisms of ER-based mechanosensing remain elusive.

We hypothesized that IRE1 might act as a sensor for mechanical perturbations of the ER. The rationale for this hypothesis is based on the observation that IRE1 has previously been shown to be activated by lipid bilayer stress, e.g. when lipid saturation is altered^12–14^. Moreover, external strain was shown to increase the tension of the ER membrane^15^. In the current work, we uncover IRE1 as an ER-resident mechanosensor. This mechanosensor activity was unrelated to the canonical function of IRE1 as a sensor of protein misfolding. Rather, IRE1 was sensing lateral membrane tension induced by mechanical perturbations. Functionally, mechano-activated IRE1 increased the level of protein translation in a manner dependent on Jun-N-terminal kinase (JNK) signaling. Finally, we show that the mechanosensing activity of IRE1 is linked to contraction-induced translation in muscle cells and serves to increase contraction strength in trained muscle cells. Our work uncovers a new, non-canonical role for IRE1 as a regulator of proteostasis in mechanically stressed cells.

## Results

### IRE1 is activated by short-term mechanical stimulation

**Figure 1:**
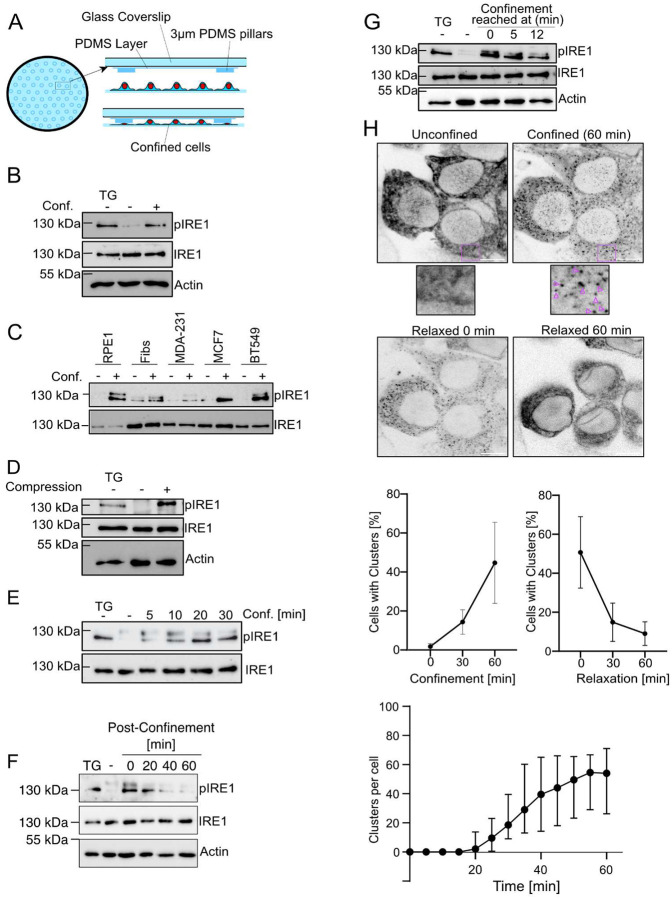
Mechanical stress activates IRE1. ***A:*** Schematic overview of the confinement method. ***B:*** HeLa cells were treated with 3 μM thapsigargin (TG) for 1 h, or cells were either not confined (−) or subjected to 3 μm confinement for 30 min (+) followed by lysis and immunoblotting as indicated. ***C:*** The indicated cell lines were either not confined (−) or subjected to 3 μm confinement for 30 min (+) followed by lysis and immunoblotting as indicated. ***D:*** HeLa cells were treated with 3 μM TG or either not treated (−) or subjected to 0.23 kPa of compression (+) followed by lysis and immunoblotting as indicated. ***E:*** HeLa cells were treated with 3 μM of TG, or either not confined (−) or subjected to different durations of 3 μm confinement as indicated, followed by lysis and immunoblotting. ***F:*** HeLa cells were treated with 3 μM of TG or were either not confined (−) or subjected to 3 μm confinement for 30 min followed by a resting period as indicated. Cells were lysed and immunoblotted as indicated. ***G:*** HeLa cells were treated with 3 μM of thapsigargin (TG) or either not confined (−) or subjected to 3 μm confinement with varying onset speed (Instantaneous, 5 min or 12 min from nonconfined to fully confined) followed by lysis and immunoblotting as indicated. ***H:*** Representative images of IRE1-GFP cluster formation/dissolving under confinement/relaxation in HeLa cells stably expressing IRE1-GFP. Purple arrows in magnified areas indicate clusters. (Top) HeLa cells expressing IRE1-GFP were confined to 3 μm for 1 h and imaged under a spinning disk confocal microscope in a 5 min interval. Confinement was then removed and cells imaged for 1 h in a 5 min interval to capture cluster dissolution. Cells with clusters were counted manually. Error bars represent SD from three experiments. (Middle) quantification of the dynamics of IRE1 clusters per cell developing over time. Cells containing clusters after 1 h of confinement were manually selected and clusters counted using Fiji. Analysis shows medians and interquartile range of 45 cells from three independent experiments (Bottom). All immunoblots in this figure are representatives of 3–4 independent experiments. Quantifications of all immunoblots are available in supplementary Table S2.

To determine whether IRE1 responds to mechanical stimuli, we performed confinement experiments with HeLa cells. Cells were confined to 3 μm for 30 min using a commercially available system based on PDMS pillars that determine the extent of confinement (Fig. 1A)^2^. Following confinement, cells were lysed and subjected to immunoblotting for phosphorylated IRE1. We observed an increase of pIRE1 under confinement, while total IRE1 levels remained unchanged (Fig. 1B). The pIRE1 antibody used in immunoblotting sometimes detected a double band. The lower band increased upon thapsigargin treatment (Fig. 1B, D–F), decreased under the IRE1 kinase domain inhibitor KIRA6 (Fig S1A-B), and was reduced after siRNA mediated IRE1 depletion (Fig S1C). However, the upper band did not respond to any of these perturbations. Therefore, we conclude that the lower band corresponds to pIRE1 and the upper band should be considered as unspecific. Phosphorylation of IRE1 by mechanical stress was prevented using KIRA6 (Fig. S1A). We observed similar results in other cell lines (MCF7, MDA-MB-231, BT549, RPE1 and human fibroblasts), indicating that IRE1 activation by mechanical stress is a general response and not restricted to HeLa cells (Fig. 1C). Phosphorylation of IRE1 was also observed when cells were subjected to compression (0.23 kPa) (Fig. 1D) or uniaxial stretching (30 min for maximally 50%) (Fig. S1D), indicating that different types of mechanical stimulation can activate IRE1. Time-course experiments showed that IRE1 phosphorylation occurred within 10 minutes of initiation of confinement and peaked at 20–30 minutes (Fig. 1E). After release of confinement, IRE1 declined after approximately 40 minutes (Fig. 1F). Mechano-activation of IRE1 is unlikely due to general toxicity of confinement because we neither observed evidence for DNA damage within 30 min of confinement nor did we observe significant cell death, even after 8 hours of confinement (Fig S1E-F).

So far, we have applied confinement instantaneously to cells, thereby inducing acute compressive stress on cells. We therefore applied confinement in a stepwise manner, reaching the final height of 3 μm after several minutes. Activation of IRE1 in response to gradual mechanical stress was substantially weaker compared to instantaneous confinement (Fig 1G).

### IRE1 forms clusters upon short-term mechanical stimulation

Active IRE1 was previously shown to form clusters when cells were exposed to proteotoxic stress^16,17^. To test whether IRE1 clusters also occur in mechanically perturbed cells, we stably expressed doxycycline-inducible IRE1-GFP in IRE1-knockout cells to avoid interference from non-tagged copies of IRE1. After induction of IRE1-GFP expression, cells were confined to 3 μm and imaged for one hour. Mechanical confinement resulted in the formation of IRE1 clusters in 44.7% of cells (Fig. 1H, Supplementary movie S1). We quantified the change in the average number of clusters per cell over time and observed that the first visible clusters appeared after approximately 10 minutes and saturated at an average value of 60 clusters per cell after about 45 minutes (Fig. 1H). Upon releasing the confinement, IRE1 clusters dissolved within 40–60 minutes (Fig. 1H, Supplementary movie S2), suggesting that IRE1 clusters are not irreversible stress-induced aggregates. Confinement led to a more pronounced cluster formation of IRE1-GFP compared to treatment with thapsigargin (TG) (Fig. S2A-C).

### Mechano-activation of IRE1 is unrelated to the UPR or to other mechanosensitive pathways

**Figure 2:**
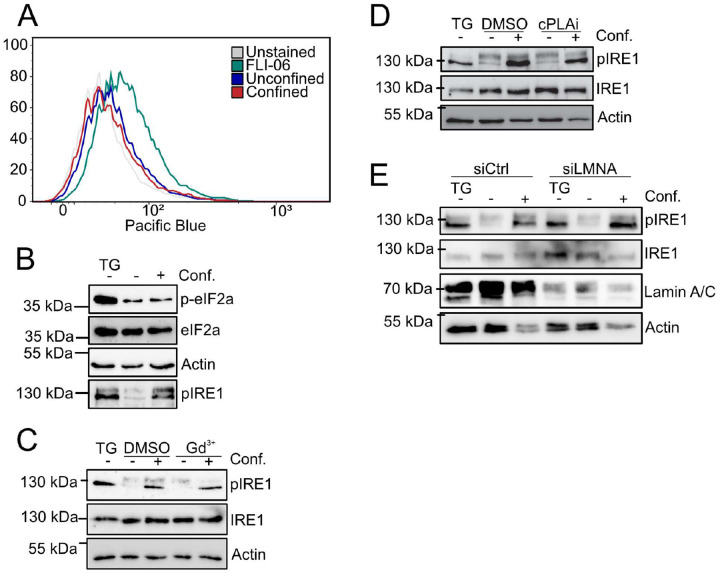
IRE1 activation under confinement happens independently of UPR and known mechanotransduction pathways. ***A:*** Representative flow cytometry graph of fibroblasts stained for unfolded proteins with TPE-MI (100 μM) either after treatment with FLI-06 (40 μM, 2 h) or during confinement (3 μm, 30 min). ***B:*** HeLa cells were treated with 3 μM thapsigargin (TG) for 1 h or either not confined (−) or confined to 3 μm for 30 min (+). ***C, D:*** HeLa cells were treated with 3 μM TG for 1 h, DMSO or gadulinium(III)-chloride hexahydrate (Gd3+, 10 μM, treatment start 15 min pre-confinement) (**C**), the cPLA2 inhibitor AACOCF3 (10 μM, treatment start 15 min pre-confinement) (**D**) and then either not confined (−) or confined to 3 μm for 30 min. ***E***: HeLa cells were transfected with the non-targeting siRNA (siCtrl) or with siRNA against lamin A (siLMNA). After 72 h, cells were either not confined (−) or exposed to 3 μm confinement for 30 min (+), followed by lysis and immunoblotting as indicated. All immunoblots in this figure are representatives of 3–4 independent experiments. Quantifications of all immunoblots are available in supplementary Table S2.

Because the canonical function of IRE1 is to sense protein misfolding, we first tested whether activation of IRE1 in confined cells is related to the unfolded protein response (UPR). To assess changes in protein misfolding, we stained cells with Tetraphenylethene-malaeimide (TPE-MI), a membrane-permeable thiol probe that was developed to detect misfolded proteins in living cells^18^. Confined cells showed no difference in TPE-MI signal compared to control cells, while treatment with FLI-06, an ER-proteostasis-disrupting agent, led to an increase in fluorescence (Fig. 2A). We also found no evidence for activation of PERK, as assessed by phosphorylation of eIF2a (Fig. 2B). We also used an ATF6 activation reporter and found that confinement does not trigger ATF6 activation (Fig. S3). Thus, brief (30–60 min) mechanical confinement of cells does not lead to measurable UPR activation.

Next, we tested whether known mechanosensitive pathways mediate IRE1 activation in mechanically stimulated cells. Treatment with gadolinium (Gd^3+^) had no effect on IRE1 phosphorylation (Fig. 2C), indicating that its activation is independent of mechanosensitive ion channels such as Piezo. We also observed no effect on mechano-activation of IRE1 by pre-treatment with trametinib (to block ERK1/2 signaling), or MK-2206 (to block Akt activity) (Fig. S4A), suggesting that IRE1 is activated independently of these mechanosensitive kinases^19–21^.

The nucleus was previously proposed to act as a mechanosensitive intracellular organelle that activates cPLA2 and induces membrane blebbing in confined cells^2,3^. Nuclear mechanosensing is likely activated under our experimental conditions, because we also observed extensive blebbing when cells were confined to 3 μm (Fig. S4B). Because the ER is intimately linked to the nuclear envelope, we tested the potential involvement of nuclear mechanosensing in IRE1 activation. Treatment with an inhibitor of cPLA2 did not affect IRE1 phosphorylation in mechanically confined cells (Fig. 2D). In addition, knockdown of lamin A, which reduces the stiffness of the nucleus^22^, did not alter confinement-induced IRE1 activation (Fig. 2E), indicating that nuclear mechanics are not directly connected to IRE1 activation under confinement. cPLA2 was also shown to be associated with higher ROS production in mitochondria during confinement^23^, but treatment with the antioxidant N-acetylcysteine (NAC) did not block IRE1 response under confinement (Fig. S4C). Thus, IRE1 activation in mechanically confined cells is unrelated to nuclear mechanosensing or cPLA2 activity. Finally, activation of IRE1 in mechanically confined cells was also independent of DNA damage, as we did not detect any measurable increase in p-yH2AX under confinement (Fig. S1E). In addition, mechano-activated IRE1 was not affected by imatinib (Fig. S4A), an inhibitor of c-Abl signaling, which was previously shown to mediate IRE1 activation downstream of DNA damage^24^. Collectively, these results imply that mechanical activation of IRE1 is unrelated to the UPR and does not involve known mechanosensitive signaling pathways.

### IRE1 is activated by direct mechanical stimulation of the ER membrane

**Figure 3:**
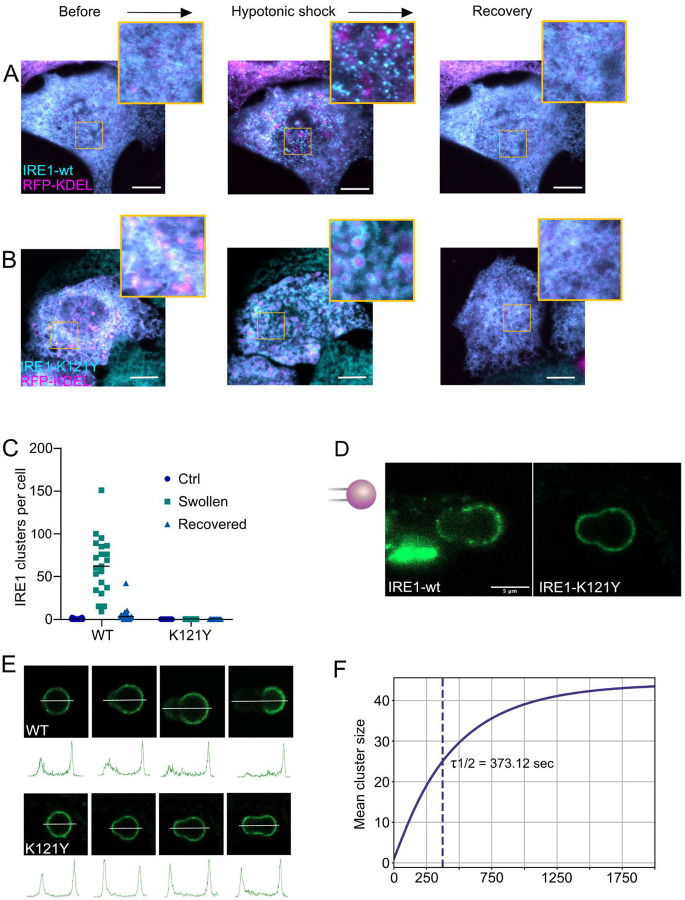
Direct activation of IRE1 by mechanical stimulation. ***A-B:*** HeLa cells expressing GFP-tagged wild-type (wt) IRE1 or a dimerization deficient mutant (K121Y) were subjected to hypotonic shock (10x dilution with water) for 20 minutes followed by recovery back to isotonic media for 20 minutes. ***C:*** Quantification of the number of IRE1 clusters per cell in the experiments from panels A&B. The number of clusters was assessed before (0 min) and 20 minutes after exposure to hypoosmotic shock. ***D-E:*** HeLa cells expressing GFP-tagged wild-type (wt) IRE1 or a dimerization deficient mutant (K121Y) were subjected to hypotonic shock. After the formation of giant ER vesicles (GERVs), the plasma membrane is ruptured to release the GERVs. Individual GERVs are subjected to direct mechanical stimulation by micropipette aspiration. Note that, contrary to the mutant, wild-type IRE1 does not distribute homogeneously along the membrane of the GERV. ***F:*** Mathematical model showing the kinetics of IRE1 cluster formation.

Our results show that IRE1 is activated in mechanically stimulated cells. However, all perturbations used so far were via external forces that act initially on the plasma membrane. To expose the ER more directly to mechanical stress, we used two complementary approaches: hypoosmotic shock of intact cells and aspiration of isolated giant ER vesicles (GERVs), a method we recently used to study intracellular membrane mechanics^25^. Using a perfusion system, we exposed cells expressing GFP-tagged wild-type IRE1 to hypoosmotic medium and assessed whether subsequent swelling of the ER would induce cluster formation. To control that IRE1 clusters occur as a result of oligomerization and not unspecific aggregation, we used cells expressing a GFP-tagged dimerization deficient mutant which has a point mutation (K121Y) in the luminal domain that impairs oligomerization^17^. The osmotic shock drives ER swelling and vesiculation, generating mechanical tension on ER membranes without applying an external force to the cell. We observed IRE1 clusters after 20 minutes of osmotic shock, indicative of IRE1 activation (Fig. 3A–C, Fig. S5). Exposing cells expressing IRE1-K121Y to hypoosmotic shock did not result in formation of any appreciable clusters (Fig. 3B–C). Returning osmotic pressure back to physiological levels dissolved the clusters, confirming their reversibility (Fig. 3A–C). To more directly expose the ER membrane to mechanical force, we used micromanipulation to rupture the plasma membrane, thereby releasing GERVs to the extracellular space^25^. Free GERVs that were not in contact with any other membrane were selected and subjected to mechanical force through aspiration into a micropipette. Mechanical perturbation of GERVs with wild-type IRE1 induced the formation of clusters (Fig. 3D), indicative of oligomerization and activation of IRE1. On the other hand, GERVs containing IRE1-K121Y did not exhibit any cluster formation (Fig. 3D). Furthermore, during micropipette aspiration of GERVs, wild-type IRE1 appeared to be excluded from the aspirated membrane tongue, while IRE1-K121Y remained evenly distributed along the membrane of the vesicle (Fig. 3E). This result indicates that IRE1 oligomers differentially distribute across membranes with heterogeneous tension or curvature levels.

Our findings support the notion that IRE1 is an ER-based mechanosensor that responds to mechanical force within minutes after stimulation. Classical mechanosensors such as Piezo channels respond to mechanical forces within milliseconds to seconds. We therefore asked why we detect IRE1 phosphorylation only after 5 minutes of mechanical confinement. IRE1 is a transmembrane kinase that must form dimers to undergo phosphorylation, which represents a rate-limiting step for detecting active IRE1. We generated a reaction-diffusion model in which 8400 molecules of IRE1^26^ diffuse within the ER membrane, where mechanical forces increase short-ranged attractive forces between IRE1 monomers leading to their clustering (see supplementary model description). Using FRAP assays, we experimentally show that IRE1 clusters are immobile (Fig. S6), and therefore implemented a size threshold whereby clusters can only grow by adsorbing smaller still-mobile clusters. The model predicted that the timescale for IRE1 cluster formation is τ1/2 = 373 seconds (Fig. 3G). Hence, full clustering of IRE1 should occur several minutes following the application of mechanical force.

### IRE1 is activated by ER membrane tension

**Figure 4:**
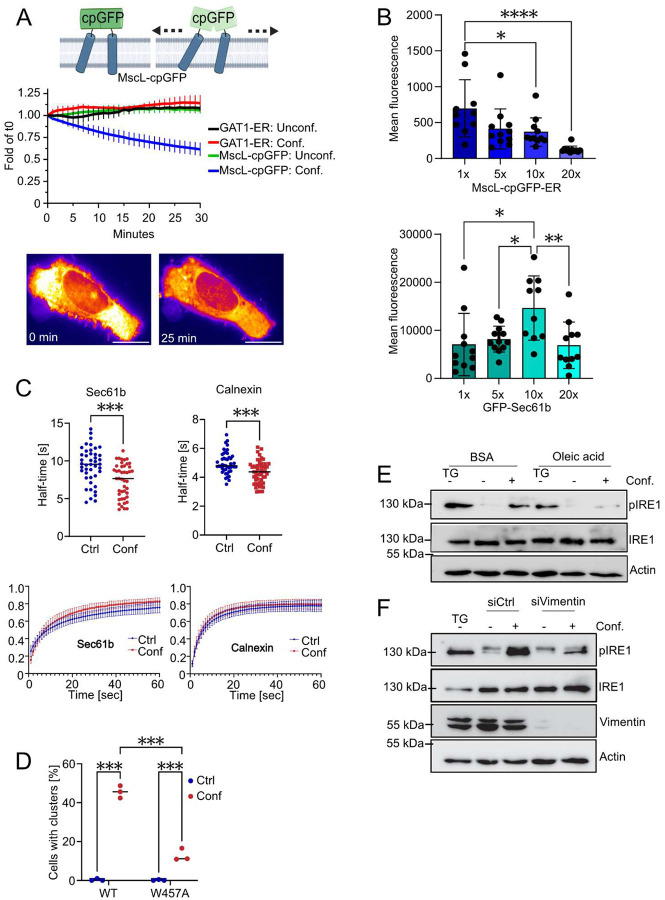
IRE1 is activated by ER membrane tension under cell confinement. ***A:*** Experiments with the MscL tension sensor show membrane tension under cell confinement. (Top) Schematic overview of tension sensing with MscL and circular permutated GFP (cpGFP). (Middle) HeLa cells transiently expressing MscL-cpGFP or GAT1-YFP were either not confined (Unconf) or confined (Conf) to 3 μm for 30 min and imaged in a 1 min interval. (Bottom) Representative images of confined cells expressing MscL-cpGFP pseudocolored to indicate fluorescence intensity. ***B:*** HeLa cells expressing MscL-cpGFP or GFP-tagged Sec61b were subjected to hypotonic shock (5x, 10x, or 20x dilution with water) for 20 minutes. Fluorescence intensity was measured before and after exposure to osmotic shock. ***C:*** Recovery halftime (Top) and recovery curves (Bottom) of fluorescence after photobleaching. HeLa cells stably expressing GFP-Sec61b (left) or Calnexin-GFP (right) were imaged under a spinning disk confocal microscope. A 1 μm^2^ area was bleached and recovery of fluorescence imaged in 1 sec intervals for 1 min, either in unconfined cells (Ctrl) or cells confined to 3 μm (Conf) for 10–30 min. p-values were calculated using Mann-Whitney-test (***p<0.001). ***D:*** HeLa cells stably expressing HA-tagged wildtype (WT) and W457A IRE1 were either subjected to 3 μm confinement or left undisturbed for 1 h before fixation and immunofluorescence staining. Cells containing clusters were counted manually. p-values were calculated using ordinary two-way ANOVA. ***E:*** HeLa cells were treated with 3 μM thapsigargin for 1 h or BSA or 200 μM BSA-Oleic acid overnight and then either not confined (−) or confined to 3 μm for 30 min (+) followed by lysis and immunoblotting as indicated. ***F:*** HeLa cells were treated with 3 μM of thapsigargin for 1 h, or either non-targeting siRNA (siCtrl) or siRNA targeting vimentin (siVimentin) for 72 h. Afterwards, cells were either not confined (−) or confined to 3 μm for 30 min (+), followed by lysis and immunoblotting as indicated. All immunoblots in this figure are representatives of 3–4 independent experiments. Quantifications of all immunoblots are available in supplementary Table S2.

Next, we tested the hypothesis that IRE1 senses changes in membrane tension that are induced by mechanical perturbations. Recent work showed that prolonged mechanical strain (6% over 6 days) increases the tension of the ER membrane^15^. However, it is unclear if short-term mechanical stress affects ER membrane tension. We used a tension sensor based on the bacterial mechanosensitive channel MscL^27^ that was engineered to contain a circularly permuted GFP (cpGFP). When lateral membrane tension increases, MscL undergoes conformational rearrangements that result in loss of fluorescence of the cpGFP molecule. We engineered this protein to localize to the ER (referred to as MscL-cpGFP for brevity) and expressed it in HeLa cells. Cells were imaged for 30 minutes before confinement to control for photobleaching. Upon confinement, we observed a marked and rapid decrease of fluorescence, indicative of an increase in lateral membrane tension in mechanically confined cells (Fig. 4A). To control whether loss of fluorescence is due to geometrical changes of the ER under confinement, we used cells expressing a fluorescently tagged, ER-localized mutant of the GABA transporter (GAT1)^28^. Confinement had no effect on the fluorescence intensity of cells expressing ER-localized GAT1 (Fig. 4A). We also tested the effect of different degrees of hypotonic shock on MscL-cpGFP and found that it responds in a graded manner, while the fluorescence of a GFP-tagged Sec61b was not affected (Fig. 4B).

To further investigate whether short-term confinement affects ER membrane properties, we performed fluorescence recovery after photobleaching (FRAP) experiments. Using cells that stably express the ER transmembrane proteins GFP-Sec61b or calnexin-GFP, we noticed that confinement resulted in an acceleration in the FRAP recovery rates of both proteins (Fig. 4C). The acceleration of FRAP dynamics is consistent with previous work showing that the diffusion of proteins in membranes is sensitive to the degree of stretching or thinning^29,30^. Together with the data obtained with ER-localized MscL-cpGFP reporter, our results support the notion that confinement affects the biophysical properties of the ER membrane.

Mammalian IRE1 was shown to utilize two residues in its transmembrane domain (W457 and S450) to sense changes in ER membrane properties in cells treated with palmitic acid^31^. To test whether W457 contributes to the ability of IRE1 to sense mechanical stress, we analyzed confinement-induced clustering of GFP-tagged IRE1 variants. Compared with wild-type IRE1, GFP-tagged IRE1-W457A showed reduced cluster formation in mechanically confined cells (Fig. 4D). To determine whether this mutation generally impairs IRE1 activation, we used prime editing to introduce the W457A mutation into the endogenous IRE1 locus in HEK293A cells (Fig. S7). These cells remained responsive to thapsigargin (Fig. S8), indicating that W457A does not abolish IRE1 activation by proteotoxic stress, but mainly altered its ability to sense mechanical stress. To further test whether membrane tension is responsible for IRE1 activation, we treated cells overnight with oleic acid, which is expected to reduce overall membrane tension. Confinement of oleic acid treated cells resulted in a clear reduction of IRE1 activation compared to BSA-treated carrier-controls, while not affecting the response of this UPR sensor to proteotoxic stress (Fig. 4E).

To gain a better understanding of the role of W457 in driving IRE1 dimerization, we used AlphaFold 3 modeling^32^ and a 30 μs long molecular dynamics simulation. The predicted model of the IRE1 dimer shows that both W457 residues are not oriented towards the interface and they are positioned opposite to each other (Fig. S9A). Thus, it is unlikely that dimer formation is driven by π-π-stacking interactions between the indol rings. This hypothesis was confirmed during the 30 μs molecular dynamics simulation, where the mean contact frequency between the tryptophan residues was just 0.13. Instead, we find that W457 forms an interaction hub and is involved in contacts with other residues in the IRE1 transmembrane helix (Fig. S9B).

Next, we asked which part of the cellular cytoskeleton is involved in transmitting the force from the cell surface to the ER. Depolymerization of the actin or microtubules network had no effect on IRE1 activation in mechanically confined cells (Fig. S10A-D). In contrast, siRNA-mediated depletion of vimentin to perturb intermediate filaments markedly reduced the response of IRE1 to mechanical stress (Fig. 4F). Altogether, these results suggest that intermediate filaments transmit mechanical forces to the ER, enabling IRE1 to sense changes in membrane tension via its transmembrane domain. Recently, external mechanical force was reported to be relayed to the ER via STIM1 at ER-plasma membrane contact sites^15^. However, silencing STIM1 did not affect the activation of IRE1 in response to mechanical confinement (Fig. S10E).

### IRE1 regulates translation under confinement

Having identified upstream mechanisms governing mechanosensing by IRE1, we next investigated which downstream signaling pathways are activated by IRE1 in mechanically challenged cells. Splicing of XBP1 is the main downstream event under conditions of proteotoxic stress. In agreement with the observation that mechano-activation of IRE1 is unrelated to the UPR, confinement did not induce any appreciable splicing of XBP1 (Fig. S11A). Accordingly, 30 minutes of confinement did not induce significant RIDD activity (Fig. S11B), indicating that mechano-activated IRE1 signals independently of its canonical RNase outputs.

**Figure 5:**
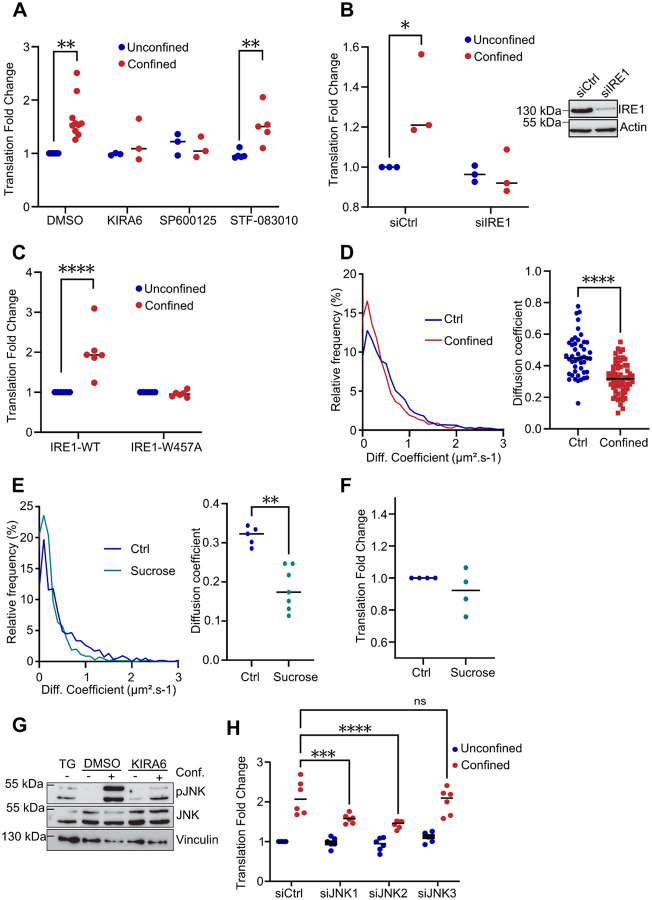
Mechano-activated IRE1-JNK axis regulates translation. ***A:*** HEK293A cells were incubated with KIRA6 (1 μM, 2 h), SP600125 (10 μM, 30 min) or STF-083010 (40 μM, 30 min) prior to confinement. Cells were treated with puromycin (10 μg/ml) for 10 min and switched to fresh medium containing the aforementioned inhibitors. Cells were then either left undisturbed or confined to 3 μm for 30 min. Puromycin incorporation was detected by anti-puromycin immunostaining and quantified via flow cytometry (SUnSET assay). ***B:*** HEK293A cells were transfected with non-targeting siRNA (siCtrl) or siRNA against IRE1 (siIRE1). After 72 h, SUnSET assay was performed in unconfined and confined (3 μm for 30 min) cells. Blot shows the efficiency of IRE1 knockdown. ***C:*** SUnSET assay was performed in wild-type HEK293A cells (IRE1-WT) or HEK293A cells with gene-edited IRE1-W457A. Unconfined and confined (3 μm for 30 min) cells were compared. ***D:*** HeLa cells stably expressing 40 nm GFP-tagged GEMs were imaged at 20 fps for 4 sec using a 60x oil immersion objective (NA 1.49). Plots show the distribution of effective diffusion coefficients. ***E:*** Distribution of effective diffusion coefficients of HeLa cells stably expressing 40 nm GEMs that were either untreated (Ctrl) or treated for 15 min with 50 mM sucrose. Imaging was performed as indicated in panel D. ***F:*** SUnSET assay was performed on HEK293A incubated either in fresh DMEM or DMEM containing 50 mM sucrose. ***G:*** Phosphorylated JNK in confined HeLa cells treated with KIRA6 (1 μM, treatment started 2 h before confinement). The immunoblot in panel G is representative of 3 independent experiments. ***H:*** SUnSET assay was performed in HeLa cells 48 h after the indicated knockdown. Unconfined and confined (3 μm for 30 min) cells were compared. Translation rate of all SUnSET Assays was normalized to control values. p-values were calculated using Wilcoxon’s signed rank test (Hypothetical median of 1. A: DMSO, B: siCtrl, C, F), or two-way ANOVA followed by Šidák’s multiple-comparisons test, showing comparisons between confined and unconfined cells (A: KIRA6, SP600125, STF083010; B: siIRE1, H) or Mann-Whitney-Test (D, E) (*p<0.05, **p<0.01, ***p<0.001, ****p<<0.001). Quantifications of all immunoblots are available in supplementary Table S2.

IRE1 was recently shown to regulate translation in an XBP1-independent manner^33^. We therefore tested whether mechano-activated IRE1 induces translation using the SUnSET assay, which measures the incorporation of puromycin into nascent proteins^34^. We used the FACS-based readout for the SUnSET assay because it is more quantitative. Confinement for 30 minutes resulted in a pronounced increase in translation, which was blocked by treatment with the IRE1 inhibitor KIRA6 (Fig. 5A). Knockdown of IRE1 similarly inhibited the induction of translation by mechanical confinement (Fig. 5B). To test whether IRE1-induced translation is related to its role in mechanosensing, we performed the SUnSET assay in IRE1-W457A cells, which are unable to sense mechanical stress. While control cells showed an approximately 2-fold increase in translation, IRE1-W457A cells were completely unresponsive to mechanical confinement (Fig. 5C). Inhibition of the RNase domain of IRE1 by STF083010 had no effect on confinement-induced IRE1 activation (Fig. S4C-D) and did not affect the increase of translation under mechanical stress (Fig. 5A).

Next, we asked how IRE1 activates translation in mechanically confined cells. Previous work showed that mechanical deformation results in volume changes that might lead to higher viscosity of the cytoplasm^35^. Therefore, we explored whether cytoplasmic crowding affects translation in confined cells. Consistent with earlier observations, we found that confinement results in a reduction in cell volume (Fig. S12A-B). Using quantitative phase imaging (QPI), we also observed that the dry mass of the cells did not change under confinement (Fig. S12C), indicating a change of density in the cytoplasm.

To determine changes in crowding and microrheology of the cytoplasm, we used cells stably expressing GFP-tagged genetically encoded multimeric microparticles (GEMs)^36^. Tracking GEM movement revealed that confinement resulted in a significant decrease in diffusivity (Fig. 5D). To determine whether the increase in cytoplasmic crowding is connected to the induction of translation, we subjected cells to hyperosmotic stress. Treatment with 50 mM sucrose mimics the degree of crowding achieved under confinement (Fig. 5E). However, hyperosmotic stress did not increase translation, but instead showed a trend towards reduced translation (Fig. 5F). This finding is consistent with previous in vitro work showing that high cytoplasmic viscosity negatively impacts translation rates^37^. We also tested whether cytoplasmic crowding might explain the absence of XBP1 splicing despite IRE1 activation in confined cells. To induce XBP1 splicing, we treated cells with thapsigargin and tested the effect of mechanical confinement. While unconfined cells exhibited robust XBP1 splicing, confinement led to a visible decrease in splicing (Fig. S11C), indicating that under mechanical confinement the biophysical state of the cytoplasm is unfavorable for efficient mRNA processing by IRE1.

It was proposed that IRE1 might control translation by binding to translation initiation factors such as eIF3B or eIF4G^33^. However, IRE1 is a low abundance protein, with copy numbers 200-fold lower than those of eIF3B or eIFG^26^. Thus, it is unlikely that IRE1 would rapidly (within 30 min) control translation via this pathway, in particular when crowding is high under confined conditions. Thus, we hypothesized that IRE1 engages an amplification mechanism to signal to the translation machinery. IRE1 is known to activate JNK^38^, a kinase that was reported to activate translation^39^. Moreover, JNK was previously shown to be activated by mechanical stress, but the mechanistic details of this activation remain poorly understood^40,41^. Confinement for 30 minutes robustly activated JNK in an IRE1-dependent manner (Fig. 5G). Notably, inhibition of JNK by SP600125 was found to blunt confinement-induced translation (Fig. 5A). To exclude potential off target effects of pharmacological inhibition of JNK, we used siRNA mediated depletion of JNK1 and JNK2 (Fig. 5H). Immunoblotting analysis showed that our cells do not express JNK3 (Fig. S13). Depletion of JNK1 or JNK2 attenuated the increase of translation under confinement (Fig. 5H, Fig. S13). Collectively, we identify a mechanosensitive IRE1-JNK signaling axis that promotes translation in mechanically confined cells. While IRE1-mediated JNK activation has typically been associated with stress-induced apoptosis^42^, our data reveal a fundamentally different functional outcome, in which mechanical activation of IRE1 engages JNK to promote anabolic protein synthesis.

### Mechanosensitive IRE1 drives translation and functional adaptation in muscle cells

**Figure 6:**
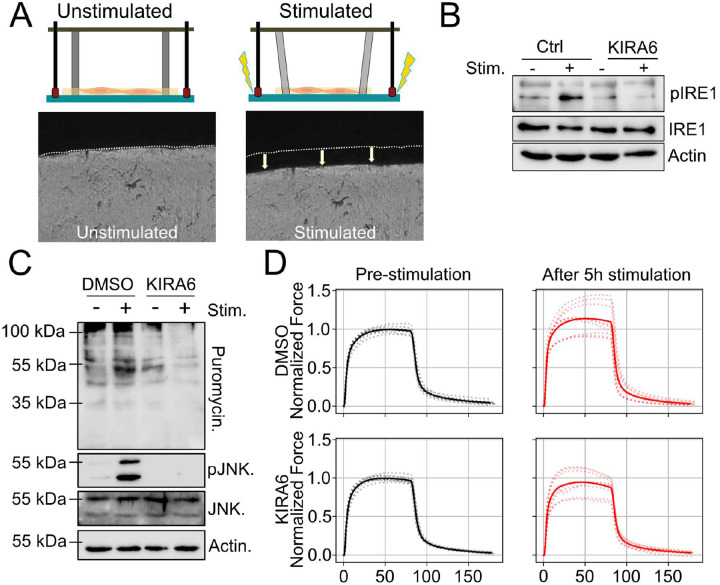
Mechanosensitive IRE1 drives translation and functional adaptation in muscle cells. ***A:*** Electrostimulation of muscle tissue. (Top) Schematic overview of experimental setup for muscle tissue stimulation. (Bottom) Post displacement under tetanic contraction. ***B:*** Muscle tissues were treated with 1 μM of KIRA6 or DMSO for 30 min before and during the following steps. Tissues were then either left undisturbed (−) or subjected to electrostimulation at 1 Hz for 30 min (+), followed by lysis and immunoblotting as indicated. ***C:*** Muscle tissues were either left undisturbed (N) or treated with 1 μM KIRA6 or DMSO for 30 min before and during following steps, and incubated with 10 μg/ml puromycin for 10 min incubation, followed by switch to fresh medium containing KIRA6 or DMSO. Muscle tissues were then either left undisturbed (−) or subjected to electrostimulation at 1 Hz for 30 min (+), followed by lysis and immunoblotting as indicated. ***D:*** Increase of exerted force in trained tissues. Tissues were treated with 1 μM KIRA6 or DMSO for 30 min before and during the experiment. Maximal force under tetanic contraction was measured by tracking post displacement under brightfield microscopy (10x magnification, NA 0.3) before and after training. For training, tissues were stimulated for 10 ms at 0.25 Hz for 5 h and 10 h. All immunoblots in this figure are representatives of 3 independent experiments. Quantifications of all immunoblots are available in supplementary Table S2.

Induction of protein synthesis in response to mechanical force is a hallmark of muscle cells, which has typically been attributed to mechanosensitive channels at the plasma membrane or the cytosolic cochaperone BAG3^43,44^. The role of mechanosensing at intracellular organelles in this response has remained unexplored. We used a previously established system^45^, in which biomimetic skeletal muscle tissue grows and self-organizes in 3D between two posts made of polymethylmethacrylate with known physical properties (Fig. 6A). Contraction of the muscle tissue results in displacement of the posts, which can be imaged using microscopy, thus allowing us to infer and quantify global forces. We first tested whether muscle contraction leads to IRE1 activation. When muscle tissue was stimulated with 10 ms electrical pulses at a frequency of 1 Hz for 30 minutes, we noticed a marked activation of IRE1(Fig. 6B) as well as JNK (Fig. 6C) that were both sensitive to the IRE1 kinase domain inhibitor KIRA6 (Fig. 6B–C). Passive stretching (5%) of muscle tissue also led to an activation of IRE1 (Fig. S14). We next performed a SUnSET assay following electrical stimulation of muscle tissue. Because the muscle fibers are embedded in a 3D fibrin matrix, we were unable to perform the SUnSET assay using FACS and therefore chose to monitor puromycin incorporation using immunoblotting. Electrical stimulation of muscle tissue strongly upregulated protein translation, which was completely blocked by inhibition of IRE1 (Fig. 6C). Although mechanical load is well known to induce translation in skeletal muscle^46^, our work is the first to implicate IRE1-based mechanosensing in this context. Finally, we tested whether IRE1-regulated translation contributes to the increase of muscle strength induced by a period of training. Force exerted by muscle tissue was measured using tetanic (5 seconds) contractions before and after a 5 hour training period (10 ms at 1 Hz). We noticed a significant increase of muscle strength after the 5 hour training period, which was completely inhibited when the training was performed in the presence of KIRA6 (Fig. 6D).

## Discussion

In this work, we establish the ER as a platform for sensing and integration of mechanical stress. We identify IRE1 as an ER-resident mechanosensor that operates independently of its canonical function as a sentinel for proteotoxic stress. Rather, IRE1 senses changes in the lateral tension of the ER membrane and converts these mechanical cues into a signaling program that regulates protein synthesis. Mechano-activation of IRE1 was observed across multiple cell types and in diverse mechanical contexts, including confinement, compression, and stretching. IRE1-mediated mechanosensing was independent of several known mechanosensitive pathways, as IRE1 remained sensitive to mechanical stress in cells treated with inhibitors of stretch-activated ion channels, ERK1/2, Akt and c-Abl. Notably, although nuclear mechanosensing was active in mechanically confined cells, ER-based mechanosensing via IRE1 operated independently of nuclear signaling, because inhibition of cPLA2 or softening of the nucleus by lamin A depletion had no impact on mechano-activation of IRE1. Thus, we propose a model in which mechanical forces are transmitted via intermediate filaments to the ER membrane, where increased bilayer tension directly activates IRE1, thereby stimulating protein synthesis in a JNK-dependent manner. We also found that IRE1 forms clusters in extracted giant ER vesicles following an increase in ER membrane tension. This provides more direct evidence that mechanical activation of IRE1 is caused by lipid bilayer tension and is not downstream of cytosolic factors. The remarkable difference in localization between wild-type IRE1 and the dimerization deficient IRE1-K121Y in aspirated vesicles indicates a selective sequestering of IRE1 oligomers in a manner dependent on membrane tension or curvature. We propose that IRE1 sequestration underlies the rapid cluster formation we observed in mechanically stimulated cells. We also identify W457 in the transmembrane helix of IRE1 as a residue critical for sensing mechanical stress. Tryptophan residues are critical for membrane anchoring^47^, but W457 has a rather unusual position being located in the middle of the transmembrane helix (position 14 out of 21 amino acids). Using in silico modeling and molecular dynamics simulations, we find that W457 is an interaction hub within the transmembrane helix, further supporting its role in dimerization and clustering. We further speculate that, located deep within the hydrophobic part of the lipid bilayer, the indol ring of tryptophan could sense lateral packing of the ER membrane and favor dimerization/oligomerization of IRE1. Recently, stress-granule-mediated phase-separation was proposed as an alternative mechanism for IRE1 cluster formation^48^. However, since IRE1 clusters also form in isolated ER membrane vesicles, we think that this mechanism is not involved in IRE1 activation. Additionally, stress granules typically form due to translational repression and eIF2α phosphorylation. In mechanically confined cells, we did not detect an increase in eIF2α phosphorylation (Fig. 2B) and instead observed a robust increase in protein synthesis (Fig. 5A–C). In addition, the IRE1-W457A mutant did not form clusters under mechanical stress, suggesting an active role for IRE1 in this process rather than a passive coalescence with stress granules.

Our time-course experiments reveal that IRE1 is activated on a scale of a few minutes. This is different from the temporal kinetics known for classical mechanosensors like Piezo channels, which operate on the scale of milliseconds to seconds. However, these slower dynamics are not incompatible with a mechanosensory role for IRE1. Firstly, IRE1 is a transmembrane kinase, thus making a comparison with an ion channel difficult. Secondly, our mathematical model shows that the requirement of oligomerization for IRE1 activation is also compatible with temporal kinetics that align well with our experimental observations. The third reason is a conceptual one: cells are exposed to highly heterogeneous mechanical inputs that differ in magnitude, duration, and temporal profile. It is therefore plausible that cells employ distinct classes of mechanosensitive systems specialized for different mechanical regimes. Rapid and transient tension fluctuations may be detected by fast-acting sensors that mediate acute adaptive responses, whereas slower or sustained increases in membrane tension may engage signaling pathways involved in longer-term cellular adaptation, including translational or transcriptional regulation. Fourth, the close temporal correlation between the ER tension measurements obtained with the MscL probe and the kinetics of IRE1 phosphorylation supports the idea that IRE1 activation is linked to progressive changes in ER membrane mechanics rather than representing a purely indirect downstream consequence of unrelated signaling events.

The identification of ER-based mechanosensing by IRE1 aligns well with earlier research on the emerging roles of the ER in mechanobiology. Mechanical forces were shown to promote ER sheet expansion, potentially via the ER-shaping protein NOMO1 or ER-PM contact sites^11,15^. Because ER-sheets are translationally more active, the rapid IRE1-driven increase in translation may represent an early adaptation to changes in ER morphology. This notion is further supported by our previous work, which showed that mechanical strain increases the rate of ER-export^5^. Increasing translation imposes an additional demand on ER calcium homeostasis to facilitate the folding of newly synthesized secretory proteins. Recent work identified SLURP1 as a modulator of the ER calcium ATPase SERCA, thereby preserving calcium homeostasis in the ER lumen^49^. SLURP1 was shown to mediate mechanical resilience of skin cells, and exploring a potential crosstalk between IRE1 and SLURP1 in skin biology could be an interesting avenue for future research. This is particularly interesting because IRE1 was also implicated in skin homeostasis^50^.

Beyond work in immortalized cells, we demonstrate that mechano-activation of IRE1 plays a part in adaptation of skeletal muscle cells. IRE1 was activated by passive muscle stretch, as well as by electrical muscle contraction and inhibition of IRE1 prevented the increase in contractile force that normally accompanies a period of repeated stimulation. Notably, the increase in translation in muscle cells was dependent on IRE1, thus potentially linking muscle hypertrophy to ER-based mechanosensing. This positions IRE1 as a hub that links mechanical load to anabolic output. These findings align with previous work reporting a cell-autonomous role for IRE1 signaling in skeletal muscle homeostasis^51,52^. Conceptually, our work extends the paradigm of mechanosensing beyond the plasma membrane and the nucleus to include the ER as an organelle capable of sensing and integrating mechanical stimuli.

Our study has some limitations. While the immunoblot experiments report activation of endogenous IRE1 under mechanical stress, the experiments on IRE1 cluster formation were performed with overexpressed GFP-tagged IRE1, even though an inducible system was chosen to have a milder overexpression. Due to the low endogenous expression level of IRE1, visualization of endogenously tagged IRE1 remains challenging. Our findings also focus predominantly on short-term (under 30 min) mechanical loading. While this was necessary to demonstrate the first ER-based mechanosensor, it imposes a limitation on the uncertainty of long-term (scale of days) mechanical loading. The SUnSET assay measures global translation and demonstrates that mechano-activation of IRE1 induces translation. However, the translational program downstream of IRE1 remains unexplored. Furthermore, the molecular mechanism of how JNK activates translation needs to be defined. Finally, our experiments on muscle contraction were carried out in engineered skeletal muscle tissues embedded in a fibrin matrix. Future work should include genetic perturbations and animal models to validate these findings at the organismal level.

## Methods

### Cell Culture

HeLa, IRE1-KO HeLa cells^53^, IRE1-KO HeLa cells reconstituted with doxycycline-inducible GFP-tagged murine IRE1^53^, HeLa cells stably expressing GFP-Sec61b or Calnexin-GFP, HEK293A cells, HEK293A cells harbouring a W457A mutation in endogenous IRE1, LentiX cells, MCF-7 cells, RPE1, MDA-MB-231 cells, and fibroblasts were maintained in DMEM (Sigma, D1145–500ML) supplemented with 10% FCS (Thermo Fisher, A5256701), 50 μg/ml Streptomycin and 50 units/ml Penicillin (Thermo Fisher, 15070063) in a humidified incubator at 37°C and 5% CO_2_. Fibroblasts were maintained in DMEM additionally supplemented with Insulin-Transferrin-Selenium (Gibco, 41400045). BT549 cells were maintained in RPMI medium containing the same additives as HeLa cell culture. All cells were tested negatively for mycoplasma by PCR in regular intervals.

AB1167 cells^54^ were maintained in Skeletal Muscle Cell Basal Medium (Promocell #C-23260) supplemented with Skeletal Muscle Growth Medium Supplement Mix (Promocell #C-39365), 15% FCS and 50 units/ml Streptomycin and 50 units/ml Penicillin and subcultured at 80% confluency until passage 16. Muscle cell tissues were seeded and differentiated as described before^45^.

siRNA mediated depletion was performed using the HiPerfect (Qiagen, #301707) transfection reagent. Per well in a 12-well plate, 10 pmol siRNA and 3 μl of HiPerfect reagent were mixed for complexing in 50 μl of serum- and antibiotic free DMEM, left to incubate for 8 min at room temperature and added dropwise to cells immediately after seeding. Experiments were performed 72h after seeding. Knockdown efficiency was confirmed either by immunoblot or by RT-qPCR if a suitable antibody was unavailable.

Plasmid transfection was performed using a 1:3 (w/w) mixture of DNA to polyethylenimine.

Oleic acid supplementation was performed by adding 200 μM oleic acid-albumin from bovine serum (Sigma, #O3008) to cell culture media and incubating cells overnight at 37 °C. Control cells were incubated overnight with bovine serum albumin solution (Sigma, #A1595).

### Plasmids

The MscL-cpGFP expression plasmid was generated by removing the sequence encoding the ER-export motif FCYENEV from MscL(G22S)61-cpGFP-ERexp (Addgene #195311) via site-directed mutagenesis PCR.

The epegRNA_IRE1_W457A construct was generated by inserting annealed spacer and 3′-extension oligonucleotides into a modified pDAS12069_U6-pegRNA backbone (Addgene #177180), after BbsI or BsmBI-v2 restriction, respectively. The backbone had been engineered to contain the tevopreq1 motif (cgcggttctatctagttacgcgttaaaccaactagaa) and to carry a puromycin resistance cassette in place of mCherry.

The ngRNA expression plasmid was generated by replacing the pegRNA cassette of pU6-pegRNA-GG-acceptor (Addgene #132777) with the sgRNA expression cassette from pSpCas9(BB)-2A-GFP (Addgene #48138) using NEBuilder HiFi Assembly, followed by insertion of annealed spacer oligonucleotides through the BbsI site.

pCMV-PE7 was a gift from Brittany Adamson (Addgene #214812).

### Prime editing

HEK293A cells were transfected with pCMV-PE7, epegRNA_IRE1_W457A and ngRNA_IRE1_W457A at a ratio of 9:3:1. The following day, cells were subjected to puromycin selection for 2 days, and multiple single-cell clones were established by limited dilution. Genomic DNA was isolated from single-cell-derived clones and the target locus was amplified by PCR using locus-specific primers. Editing was initially assessed by Sanger sequencing and subsequently confirmed by amplicon-based nanopore sequencing (Plasmidsaurus). In the clone used throughout the manuscript, all detected *ERN1* alleles carried the intended W457A edit. Of note, one of the three detected alleles additionally contained an I462S substitution. No other unintended substitutions, insertions or deletions were detected in the ~350 bp amplicon window.

### Microscopy

GCampER experiments, IRE1 cluster assays, calcein cell volume experiments, MscL tension sensor experiments and fluorescence recovery after photobleaching experiments were performed on a Nikon Ti2 microscope with a spinning disk confocal system (Crest V2), using a 60x oil immersion objective (CFI Apochromat TIRF, NA 1.49).

### Confinement

Cell confinement was performed in the 4Dcell confinement system. For biochemical analysis, 150,000 HeLa cells or 100,000 HEK293A cells were grown on 18 mm coverslips overnight. If necessary, compounds were added for a pre-incubation period (for concentration and pre-incubation time, consult Table S1). For confinement, cells on coverslips were transferred to the 4D cell static confiner with 500 μl of medium. The confiner was pre-incubated for 5 min at culture conditions with the lid placed loosely to equilibrate the PDMS pillars of the confinement slides in medium. Then, the confinement was set and incubated at culture conditions for 30min. After opening the confiner, the coverslip was carefully detached from the confinement slide, washed by dipping in ice-cold PBS, and cells scraped off in NP-40 lysis buffer (50 mM Tris-HCl 7.4 pH, 150 mM NaCl, 1% NP-40).

For confinement during life cell imaging, 300,000 cells were grown in 35 mm glass-bottom dishes (Ibidi) overnight. Before imaging, the dish was placed into the Tokai life cell chamber set to 37 °C and 5% CO2. The pump (Elveflow, Cobalt autonomous microfluidic pump) attached to the 4D cell dynamic confiner was set to −15 mbar and the suction cup placed over the imaging field. To set confinement, the pump was set to −150 mbar.

For slow confinement, the pump was set to −70 mbar (no contact between cells and confinement slide) and the pressure manually decreased to −150 mbar at different speeds (Instantaneous, 60 mbar/min for 5 min, 5 mbar/min for 12 min) using a stopwatch. After 30 min of full confinement, confinement was released, cells from the imaging field washed with PBS and scraped off in NP-40 lysis buffer.

### Imaging of IRE1 clustering

IRE1-KO cells were reconstituted with a doxycycline-inducible IRE1-GFP using lentiviral transduction and subsequent FACS sorting for positive cells. IRE1-GFP cells were seeded 48 h prior to the experiment in Ibidi life cell imaging dishes and treated with 100 nM of doxycycline for 16–24 h to reach visible levels of IRE1-GFP expression. Cells were imaged on a Nikon spinning disc microscope with a 60x oil objective. Images were acquired at 1 min intervals for 1h.

### Cluster Counting

IRE1-GFP clusters were counted using Fiji. Cells containing clusters after 1 h of confinement or thapsigargin treatment were selected using the freehand selection tool and duplicated. A Laplacian of Gaussian filter was applied using the LoG3D plugin^55^ to increase edge detection. For measuring cluster size, thresholds were set using the MaxEntropy pre-setting. For detecting cluster development over time, the Triangle pre-set was used because it yielded better results at detecting clusters with high ER background fluorescence. The threshold was manually adjusted to represent cluster distribution in original images and prominent artefacts (i.e. edge of the cell or edge of the nucleus) manually removed. Clusters were counted using the ‘Analyze Particles’ function.

### Cell Compression and Stretching

For compression, 150,000 HeLa cells were grown on 18 mm coverslips overnight. Plugs of 2.4% agarose were made with serum- and antibiotic-free DMEM in a 12-well plate. After removal from the plate, the plugs were UV sterilized and pre-warmed to 37 °C. We calculated the compressive force of a plug (weight 6 g) on the area of the coverslip (2.54 cm^2^) to be ~0.23 kPa. The coverslip was then placed on parafilm and a plug set on top. Complete DMEM was pipetted around the base of the plug to prevent drying. Compression was carried out for 30 min at 37 °C.

For stretch experiments, the surface of PDMS uniaxial stretch chambers (Strexcell, SC-0040) were coated with poly-L-lysine (Sigma-Aldrich, P4832) according to the manufacturer’s instructions. Fibroblasts (50,000 cells per chamber) were seeded in complete growth medium. After 24 h, cells were subjected to 50% uniaxial stretch for 1 h. Unstretched chambers and chambers treated with thapsigargin (2 μM, 1 h) were included as controls. Then, chambers were washed with ice-cold PBS and placed on ice. Cells were lysed, and analysed by immunoblotting for phospho-IRE-1, total IRE-1, phospho-JNK and actin.

### Fluorescence Recovery after Photobleaching

FRAP experiments were carried out on a Nikon spinning disc microscope (Crest V2) using a 60x oil immersion objective. A 1×1 μm field was bleached for 500 ms using 50% laser intensity. Recovery was imaged in spinning disk mode in a 1 sec interval for 1min. Image analysis was performed in ImageJ and recovery halftime calculated using GraphPad Prism 10 (One Phase Association).

### SUnSET assay

After pre-incubating with the respective compounds, HEK293A cells were incubated with 10 μg/ml of puromycin for 10 min at culture conditions. Cells were then transferred to pre-incubated fresh DMEM in confiners or cell culture plates and confinement performed as described above. After confinement, cells were detached by gentle pipetting in ice cold PBS. Cells were prepared using the BD Cytofix/Cytoperm kit, fixed for 30 min and stained for 45 min with an anti-puromycin antibody labeled with Alexa Fluor 647. Samples were measured with a Symphony Cell analytics Flow Cytometer using the R670/50 channel.

### TPE-MI Assay

150,000 HeLa cells or 100,000 human fibroblasts were seeded on 18 mm coverslips and left to adhere overnight. Positive controls were incubated with 40 μM of FLI-06 or 6 μM of thapsigargin for 2–3 h. Cells pre-incubated with staining solution (serum free DMEM, 100 μM TPE-MI dye, 1% DMSO) for 5 min at culture condition and then confined in staining solution for 30 min. After release of confinement, cells were detached with trypsin and collected by centrifugation. TPE-MI signal was detected using the PacificBlue channel of a LSR Fortezza Flow Cytometer with a 405 nm laser and a 450/50 filterset.

### Imaging GEMs to infer cytoplasmic crowding

HeLa cells stably expressing cytosolic GEMs were generated by lentiviral transduction. Cells expressing similar expression of GEM particles were imaged in widefield fluorescence microscopy (Nikon Ti2) at 20 fps for 4 sec using a 60x oil immersion objective (NA 1.49). After imaging of unconfined cells, the cells were confined with the dynamic confiner as described above. Confined cells were imaged after 15 min of confinement. Data analysis was performed as previously described^36^.

### Immunoblots

After SDS-PAGE, proteins were transferred to a nitrocellulose membrane and subsequently blocked in fresh Rotiblock solution overnight at 4 °C. For primary antibody dilution and incubation conditions, consult Table S1. After 3×3 min washing in TBST, the blots were incubated with secondary HRP-tagged antibodies (1:8000 in 5% milk TBST) for 2–6 h. To prevent stripping artefacts when detecting IRE1 and phosphorylated IRE1, samples were loaded on two blots and incubated in one antibody each.

### RIDD and XBP1 Splicing Assay

After confinement or thapsigargin treatment, total RNA was extracted using a NEB Monarch RNA extraction kit, following the protocol provided by the manufacturer. One μg of RNA was transcribed to cDNA using NEB LunaScript Mastermix. For XBP1 splicing assay, XBP1 and GAPDH were amplified with Taq Polymerase (GoTaq, Promega) following the manufacturer’s instructions (annealing temp. 55 °C). Primers were described before^56^. XBP1 splicing was visualized by running samples on a 2–3% agarose gel. For the RIDD assay, qPCR reactions were performed with Luna qPCR mastermix (NEB) to amplify the RIDD target Bloc1S1 in a StepOnePlus qPCR machine (Applied Biosystems, Thermo Fisher Scientific).

### Micromanipulation

To aspirate and manipulate cells, glass capillaries (1.0 mm OD × 0.58 mm ID × 150 mm; GC100–15b, Harvard Apparatus) were pulled into micropipettes on a laser puller (P-2000, Sutter Instruments) using a standard program (Heat 580, Filament 3, Velocity 50, Delay 130, Pull 180) to obtain tip diameters of 3–5 μm. The pipette tips were excised with an MF2 microforge (Narishige Group), plasma-cleaned and treated with a PEG-based solution (3 mg/ml; Cat#JKA3037 from Merck) prepared in ethanol:H2O (95:5 v/v). Then, micropipettes were cleaned with DMEM: H2O (5:95 v/v).

Cell manipulation and aspiration were carried out using a motorized micromanipulator mounted to the microscope stage, with samples maintained at 37 °C in hypotonic DMEM.

### Giant ER vesicles (GERVs) formation extraction and imaging

To isolate giant endoplasmic reticulum vesicles (GERVs), swollen cells were subjected to gentle pipetting to shear them, with at least 4 suction-and-refill cycles. This process lysed the cell’s plasma membrane, leading to the release of GERVs. Before micromanipulation, biological samples were pipetted onto a coverslip glass plate, pre-treated with BSA, and washed with DMEM: H2O (5:95 v/v). Under the microscope, GERVs were gently caught with the micropipette. Tension was increased steadily (~ 2 mbar every 30 seconds) while performing a time-lapse to observe the behavior of the membrane.

All images were taken on a Carl Zeiss LSM 800 (Carl Zeiss, Oberkochen, Germany) with a 63× oil-immersion objective, and the samples were held on glass coverslips (24 × 36 mm #0; Menzel Gläser, Braunschweig, Germany).

### Cell volume measurement

Cells were pre-incubated with Calcein-AM (0.1 μM) in serum-free media for 60 min at 37 °C followed by a change to fresh complete media. Calcein fluorescence was measured by widefield microscopy live imaging on a Nikon Ti2 microscope using a 60x oil immersion objective (CFI Apochromat TIRF, NA 1.49). Images were recorded at 15 min intervals for 30 minutes. Volume was calculated by subtracting the background and normalizing by the unconfined condition.

### Quadrilateral wave shearing interferometry

Quantitative phase imaging was carried out on a Zeiss AxioObserver microscope (Carl Zeiss, Oberkochen, Germany) controlled by VisiView software (Visitron Systems, Puchheim, Germany) using a Zeiss 63x Plan-Apochromat objective. Sparsely seeded cells were grown on glass bottom dishes and were either left untreated or confined to 3 μm for 30 minutes. Cells were fixed for 20 minutes in paraformaldehyde washed and imaged in PBS. Images were taken using a Phasics SID4 1550 wavefront sensor camera controlled by SID4Bio software (Phasics, Saint-Aubin, France). Reference images from empty areas were used to calculate the optical path length and dry mass using SID4Bio software.

### Membrane tension measurements

Cells grown on glass-bottom dishes were transfected with a plasmid encoding MscL-cpGFP. On the next day, cells were imaged by widefield live imaging on a Nikon Ti2 microscope using a 60x oil immersion objective (CFI Apochromat TIRF, NA 1.49).

### AlphaFold 3 model and Molecular Dynamics simulations

To predict the structural basis of IRE1 homodimerization at the level of the transmembrane (TM) helix, we used AlphaFold 3 (AF3) modeling^32^ with custom, species-specific multiple sequence alignments (MSAs). We obtained ortholog sequences for the protein from the NCBI Gene Orthologs database (accessed 15 March 2026). These sequences were then reduced to one per species with the highest identity to the Homo sapiens reference sequence using the “best-reciprocal-hit” strategy of the EVcomplex framework^57^. This yielded 5,085 species-matched pairs. Rather than modelling the full-length protein as a homodimer, we used the truncated sequences (amino acids 440–500) that include the TM helix as an input to build the custom paired MSAs. We performed complex structure predictions using a local AF3 installation with pre-defined weights, five random seeds (25 total models) and custom paired MSAs, bypassing the default AF3 pipeline. The models were ranked by the AF3 ranking score.

The top-ranked model from each seed was selected for structural superimposition to evaluate interface convergence. Superimposition and visual analysis were performed in PyMOL (Schrödinger, LLC). We examined predicted aligned error (PAE) maps, per-residue pLDDT (Predicted Local Distance Difference Test) scores, and inter-chain heavy-atom distances from predicted structures to access the confidence in the local helix geometry.

The predicted homodimer structure was truncated to the region predicted as TM helix in the Uniprot Database^58^ and its neighboring residues (amino acids 440–467). The truncated homodimer structure was prepared and protonated using MOE (Molecular Operating Environment, version 2024.06, Molecular Computing Group Inc, Montreal, Canada). A membrane-protein system was built using CHARMM-GUI^59^. The PPM 2.0 web server^60^ was employed to position the dimer in a 65Å*65Å lipid bilayer composed of 65% POPC, 35% POPE and 10% cholesterol. A 22.5Å aqueous layer was added on each side of the membrane. Molecular Dynamics simulations were conducted using Amber24^61^. The system was parametrized with LEaP from AmberTools24^62^. ff14SB was used as the protein force field, while the lipids were parametrized using Lipid21^63,64^. TIP3P was chosen as the water model^65^. The system was minimized by minimizing gradually all hydrogen atoms, heavy atoms of water, lipids and protein. We then performed equilibration by heating the system from 100K to 300K, minimizing to cool down, and heating it up to 300K again. Finally, we further equilibrated the system with 1ns isotropic position scaling, followed by 10 ns pressure scaling. After equilibration, the production run was conducted at 300K and atmospheric pressure. The Langevin thermostat was used to control the temperature and the Berendsen barostat was used to control the pressure. 10 independent replicas of 3 μs each were conducted. The analysis of the interactions between the two helices was conducted using GetContacts^66^. The contacts are determined based on the default geometrical criteria provided by GetContacts. The analysis was conducted on 150000 frames (1 frame every 200 ps).

### Muscle tissue sample preparation

Muscle tissues were treated with 1 μM of KIRA6 or 2 μl/ml DMSO and pre-incubated for 30 min. Tissues were stimulated to twitch using electrical pulses (1 Hz, Artificell T-Pulse) for 30 min and subsequently harvested with tweezers and washed in 1 ml of ice-cold PBS. The tissues were homogenized in a dounce homogenizer under addition of NP40 lysis buffer and the samples centrifuged for 15 min at 21000 rcf. Immunoblotting was performed as described above.

For SUnSET assays, the tissues were incubated for 10 min with 10 μg/ml puromycin, after which the tissue culture medium was replaced with fresh medium before stimulation as described above.

### Muscle tissue force measurement

Muscle tissues were pre-treated with KIRA6 or DMSO as described above. Videos for force measurement were acquired in brightfield (Nikon Ti2) using a 10x objective (NA 0.3) at 50 fps. Tissues were stimulated using a 5 second electrical pulse to induce a tetanic contraction. Prior to acquiring images, tissues were subjected to 10 Twitches (1 Hz) and 3 tetanic contractions. For training, cells were stimulated for 10 ms at a frequency of 0.25 Hz for 5 h and 10 h.

The force curves are derived by tracking the displacement of the pillars throughout the tissue contractions with a self developed algorithm. The algorithm essentially searches for the displacement that minimizes the negative normalized cross correlation between the first frame and every consecutive frame. The minimization was performed with the Cross-Entropy-Method^67,68^. To derive the forces from the deflection the previously determined spring constant was used as in here^45^.

For each tissue three tetanus contractions were obtained before and after the five hours of training. To take into account the tissue to tissue variability the forces were normalized with their respective average peak force before the stimulation.

### Muscle tissue passive stretch

To apply passive mechanical stress to the muscle tissues, the cultivation system was modified to accommodate a right-angled bar between the two pillars in each well (see SI). The bar was attached to a Thorlabs Piezo Inertia actuator (Cat No: PIA25) and positioned against one of the pillars, causing the tissues to be elongated by approximately 15% of their initial length of 4 mm. This mechanical stress was maintained for 30 minutes at a constant temperature of 37 °C. Following the stress application, the tissues were transferred to 2 ml Eppendorf tubes containing 200 μl of ice-cold NP-40 lysis buffer supplemented with phosphatase and protease inhibitors. The tubes were then snap-frozen in liquid nitrogen and stored at −80 °C. Prior to western blotting, the samples were mechanically disrupted using a TissueLyser III (Qiagen).

### Statistics

The number of experiments and the number of cells used for generating data are indicated in the respective figure legends. All statistical analysis was performed in GraphPad Prism 10 (Version 10.2.3).

### Mathematical modeling of IRE1 clustering

Details about the model are provided in the supplementary model description.

## Supplementary Material

Supplementary Files

This is a list of supplementary files associated with this preprint. Click to download.

- TableS1new.xlsx
- TableS2new.xlsx
- SupplementarymovieS2.avi
- SupplementarymovieS1.avi
- Supplement.pdf
